# The location of cemento enamel junction for CAL measurement: A clinical crisis

**DOI:** 10.4103/0972-124X.51888

**Published:** 2009

**Authors:** K. L. Vandana, Ira Gupta

**Affiliations:** *Senior Professor, Department of Periodontics, College of Dental Sciences, Davangere, Karnataka, India*; 1 *Graduate Student, Department of Periodontics, College of Dental Sciences, Davangere, Karnataka, India*

**Keywords:** Clinical attachment level, cemento-enamel junction, close measurements, open measurements, stent

## Abstract

**Background:**

We face various problems while measuring the Clinical attachment level (CAL) from Cemento-enamel junction (CEJ). This study aims to record and compare the CEJ location measurements using a xed reference point (FRP) [Custom made stent] before and after ap elevation.

**Materials and Methods:**

A custom made stent and UNC-15 probe were used. Recording of CEJ location was made using a UNC-15 (Hu-Friedy) probe, before (close CEJ) and after (Open CEJ) the reflection of the flap from the lower edge of the stent in those subjects who were indicated for flap surgery, at baseline.

**Results:**

We used statistical analysis involving intra-group comparison done by Paired-‘t’ test. The close and the open CEJ measurements demonstrated a, statistically, non-significant difference. The equi-measurements of close and open CEJ numerical data were remarkably lower than the under and overestimation of measurements. Thus, despite certain disadvantages of stent, the FRP provides a simple solution for CAL measurement.

**Conclusion:**

The results of this study confirms the objective of the study and strongly suggests that CAL measurements done without FRP is subjected to great variation and the diagnostic and prognostic interpretation of CAL should be viewed seriously in periodontics.

## INTRODUCTION

One of the most important parameters to assess periodontal destruction is loss of connective tissue attachment to the tooth root surface. The Cemento-enamel junction (CEJ) acts as a static landmark to measure CAL or periodontal destruction.[1] Thus, the CEJ is a very important landmark for the periodontist, but various problems are faced while measuring CEJ, as difficulty in probing specially when it is subgingival[2] and it is difficult to detect CEJ if obscured by calculus or dental restorations.[3] Also, the three hard tissues found at the CEJ region are unpredictable and irregular on a single tooth and contra lateral teeth.[4]

In the past, recording of pocket depths was the only method to measure periodontitis. However, merely probing the pocket and recording the pocket depth is insufficient as increase in pocket depth could be due to a gingival enlargement without destruction of underlying periodontal tissue. In some cases, recession of marginal gingival may accompany attachment loss, thus masking ongoing disease progression if the pocket depth measurements are taken alone.

If the CEJ is not present due to a restoration, or difficult to determine, it can be recorded from margin of a restoration or a stent and the recording thus made is referred as Relative attachment level (RAL).

The assessment of attachment levels due to its fixed reference point (FRP) provides better information relating to gain or loss of attachment to the root surface and in assessing the disease progression as compared to pocket depth measurements. Though attachment level measurements too have certain inherent shortcomings like its high level of tactile sensitivity and time consumption, it still represents “Gold Standard” by which clinicians record disease status.[3]

There are several studies discussing reliability of CAL using CEJ and FRP in assessment of periodontal therapy; however Medline search reveals no data on location of CEJ using stent before and after reflection of flap.

The routine assessment of CAL is subjected to considerable over or under estimation due to problems associated to exact location sub-gingival location of CEJ, the non-visibility and tactile error are the major setback in identifying true CEJ levels. To determine the CAL measurement error, the prime objective of this study is to locate CEJ before (close) and after (open) flap reflection using a fixed reference point so as to ascertain the number of times the close CEJ was under, over or equi-estimated with the open CEJ measurements.

## MATERIALS AND METHODS

For this study, subjects belonging to both the sexes were selected from the outpatient department of Periodontics, College of Dental Sciences, Davangere, Karnataka, India. The age group of the patients selected ranged from 16 to 45 years. The inclusion criteria was - patients who required periodontal flap surgery. The exclusion group consisted of patients with a history of known systemic diseases/pregnant and lactating women. Total no. of sites measured was 232.

### Recording of CEJ

A custom made stent and UNC-15 probe were used. The stent was made with the cold cure acrylic by the sprinkle method. It covered the occlusal/incisal 1/3^rd^ on the buccal and the lingual side.[5] The thickness of the stent was about 2-3 mm. The vertical grooves were made on the stent on buccal and lingual side using straight fissure bur no. 556 and air-rotor handpiece to guide the UNC-15 probe at selected sites. The stent was made to fit on the occlusal/incisal surfaces of the teeth and the measurements were made using the UNC-15 probe by placing it in the groove made on the stent. Recording of CEJ location was made using a UNC-15 (Hu-Friedy) probe, before (Close CEJ) and after (Open CEJ) the reflection of the flap from the lower edge of the stent in those subjects who were indicated for flap surgery, at baseline. The same investigator recorded all the measurements to the nearest 0.5 mm. The measurement data thus collected at different time periods were subjected to statistical analysis. Paired-‘t’ test was used to make intra-group comparison of measurements for CEJ, before and after reflection of flap.

## RESULTS

Comparison of the measurements of close and open CEJ locations, i.e., before and after the reflection of flap was made [Tables 1–5]. The mean and standard deviation of close and open CEJ measurements was 5.72plus/minus1.43 and 5.60 plus/minus 1.38 respectively. The difference was found to be statistically not significant (p equal to 0.07) between the two measurements. While comparing the individual measurements it was seen that 25.4 % of closed and open measurements did not differ. However, 43.6% were underestimated which included maximum measurements (33.6%) to be underestimated by 0.5 mm. 31.1 % measurements were overestimated.

**Table 1:** Comparison of close and open measurement of CEJ

	Close CEJ	Open CEJ	Diff.	t-value	*P*-value*
Mean	5.72	5.60	0.11	1.8	0.07, NS
SD	1.43	1.38	0.95	-	-

* Paired-‘t’ test, NS - Non significant

**Table 2:** Distribution of the number and percentage of sites of close and open CEJ at different levels

Diff. (mm)	No. of sites	Percentage (%)	
−2	2	0.9	
−1.5	3	1.3	
−1	18	7.8	43.6%
−0.5	78	33.6	
0	59	25.4	
0.5	19	8.2	
1	28	12.1	
1.5	7	3	
2	10	4.3	
2.5	3	1.3	31.1%
3	3	1.3	
3.5	2	0.9	

Total no. of sites measured = 232.

**Table 3:** Comparison of CEJ measurements by using close and open method (mean ± SD)

Sites	CEJ measurement		t - value	*P* value	Significance
				
	Open method	Close method		
Midbuccal N = 46	6.02±1.64	6.17±1.50	0.464	0.64	NS
Midlingual N = 45	5.55±1.14	5.43±1.17	0.500	0.62	NS
Mesio buccal N = 46	5.67±1.42	5.72±1.58	0.138	0.89	NS
Disto buccal N = 44	5.51±1.35	5.62±1.39	0.356	0.72	NS
Mesiolingual N = 46	5.30±1.29	5.5±1.47	0.676	0.50	NS
Distolingual n = 6	5.5±0.44	5.83±0.75	0.932	0.37	NS
All sites n = 232	5.61±1.37	5.69±1.43	0.630	0.53	NS

*P* < 0.05, NS - Non significant.

**Table 4:** Comparison of differences in CEJ measurements of buccal sites using open and close methods (percentage)

Diff. in measurements	Mesio buccal site	Percentage reduction		Disto buccal sites	Percentage reduction		Mid buccal	Percentage reduction	
−4.0	0	0	23.92	0	0	33.33	0	0	32.60
−3.5	1	2.17		0	0		0	0	
−3.0	1	2.17		1	2.38		0	0	
−2.5	0	0		0	0		2	4.35	
−2.0	3	6.52		2	4.76		3	6.52	
−1.5	1	2.17		1	2.38		2	4.35	
−1.0	4	8.69		7	16.66		4	8.69	
−0.5	1	2.17		3	7.14		4	8.69	
0	8	17.39	17.39	9	21.42	21.42	12	26.08	26.08
0.5	20	43.47	58.69	14	33.33	45.23	14	30.43	41.30
1	6	13.04		3	7.14		4	8.69	
1.5	1	2.17		1	2.38		0	0	
2	0	0		1	2.38		1	2.17	
2.5	0	0		0	0		0	0	
3	0	0		0	0		0	0	
3.5	0	0		0	0		0	0	

**Table 5:** Comparison of differences in CEJ measurements of lingual sites using open and close methods (percentage)

Diff. in measurements	Mesio lingual site	Percentage reduction		Disto lingual site	Percentage reduction		Mid lingual	Percentage reduction	
−4.0	0	0	26.08	0	0	50	0	0	22.22
−3.5	1	2.17		0	0		0	0	
−3.0	1	2.17		0	0		0	0	
−2.5	0	0		0	0		0	0	
−2.0	3	6.52		0	0		0	0	
−1.5	1	2.17		0	0		2	4.44	
−1.0	3	6.52		2	33.33		4	8.88	
−0.5	3	6.52		1	16.66		4	8.88	
0	13	28.26	28.26	2	33.33	33.33	13	28.88	28.88
0.5	18	39.13	45.65	1	16.66	16.67	17	37.77	48.88
1	3	6.52		0	0		5	11.11	
1.5	0	0		0	0		0	0	
2	0	0		0	0		0	0	
2.5	0	0		0	0		0	0	
3	0	0		0	0		0	0	
3.5	0	0		0	0		0	0	

The comparison of Open V/s Close CEJ measurements at individual sites revealed no significant difference between the methods. The discrepancy of measurement at different levels for various sites demonstrated higher % of overestimated values for mesiobuccal, distobuccal, midbuccal, mesiolingual and midlingual sites. For six distolingual sites, a higher % of the close CEJ measurements was underestimated (50%). On the whole, the equi-measurements of close and open CEJ numerical data was remarkably lower than the under and overestimation of measurements [Tables 3–5].

## DISCUSSION

The assessment of attachment levels provides better information relating to gain or loss of attachment to the root surface and in assessing the disease progression as compared to pocket depth measurements.

The recording of CAL using close CEJ measurements is considered to estimate risk and progression of periodontitis; as well the outcome of periodontal therapy. The relative attachment level is an alternative to CAL and not a substitute as the FRP is always coronal to CEJ and the true attachment level measurements are from CEJ to base of the pocket (BOP). The RAL measurements are good enough in non-surgical periodontics. However, the CAL measurements are ideal for surgical periodontics wherein it is convenient to record CEJ during flap exposure. The FRP provided by the custom stent serves as a good tool to understand the over/under estimation of CEJ location before and after flap reflection.

There are several papers discussing reliability and validity of CAL following periodontal therapy, however, the lack of data on the comparison of close versus open CEJ measurements prompted the present study.

The open CEJ measurements are the true measurements as the location of CEJ is visualized after flap reflection. On comparison of close and open measurements of CEJ i.e. the measurements made before and after the reflection of the flap, different levels of differences was seen between the two measurements. With regard to mean values and variability of the measurements, there was no significant difference between close and open measurements. In terms of statistics, the non significant difference between open and close CEJ measurements could be due to nullifying effect of overestimated and underestimated values of close CEJ measurements. This can be appreciated by the fact that out of the total 232 sites examined, the close CEJ measurements were overestimated by 31.1% (72 sites) and underestimated by 43.6% (101 sites) and only 25.4% (59 sites) matched equally (without any difference). The comparison of open Vs close CEJ measurements at individual sites revealed no significant difference between the methods. The discrepancy of measurement at different levels for various sites demonstrated higher percetage of overestimated values for mesiobuccal, distobuccal, midbuccal, mesiolingual and midlingual sites. For six distolingual sites, a higher per cent of the close CEJ measurements was underestimated (50%). On the whole, the equimeasurements of close and open CEJ numerical data was remarkably lower the under and overestimation of measurements [Tables 3–5]. The results of this study confirms the objective of the study and strongly suggests that CAL measurements done without FRP is subjected to great variation and the diagnostic and prognostic interpretation of CAL should be viewed seriously in periodontics. Although the results of the present study show that difference between close and open measurements of CEJ are not significant, the descriptive analysis of various underestimated and overestimated measurements of close CEJ is self explanatory. The interpretation of the findings have to be seen in the light of biological (clinical) significance where a narrow range error may be necessary for specific research objectives.

A custom-made plastic occlusal stent used in this study measures attachment levels in lieu of the traditional approach which uses the cementoenamel junction (CEJ) as a fixed reference point. Bulk to the stent on the occlusal surface contributes to its strength. It also makes sense to inscribe a line on the master cast at the apical edge of the stent; therefore if the stent is lost or broken, a new one can be fabricated. While this is likely to create some error, it is better than losing the data altogether.

The reproducibility of periodontal measurements is better using a stent. Also in a study by Watts T, where four sets of measurements were performed for the gingival margin, probing depth and CEJ by an examiner using a constant force probe with and without stent; stent to CEJ showed the least exact reproducibility (53%) of the simple measurements.[6] In another study by Watts T.L.P[7] where the comparative probing reliability with recession and CEJ measurements were studied using two different kind of probes; the results showed that the two duplicate measurements of stent to gingival margin did not differ significantly, but the differences were apparent in the CEJ measurements.[8] Isidor *et al,*[9] recommended the use of flexible splints to produce fixed reference points and reproducible measuring sites, this allows pocket depths and attachment levels to be measured with a reproducibility of one mm.

One electronic probe with the capability to detect the CEJ has been described, but this probe has been primarily used as a research tool only.[10] In contrast, the Florida probe[11] is a third generation, commercially available, periodontal probe that combines controlled force application, automated measurement and computerized data collection and provides a means of recording attachment changes over time. When using the Florida probe, attachment levels are recorded relative to a fixed reference point, for example the occlusal surfaces of the teeth or a pre fabricated stent.[12] Unfortunately, the use of relative reference points provides no information relating to attachment levels at a single examination, and furthermore, the reference points may change (a tooth may be restored or a stent may become distorted). For these reasons, it is desirable to measure clinical attachment levels using the CEJ as the reference point. However, the difficulties of reproducibly locating the CEJ have induced investigators and clinicians to select other reference points for the measurement of relative attachment levels.[6–11]

Future studies should concentrate on recording of interproximal CEJ which is highly variable and diffcult to measure.

## CONCLUSION

Periodontal therapy is often based upon information regarding longitudinal attachment level data. Accurate, reliable measurement of attachment level is an important aspect of periodontal diagnosis and recommended treatment. If periodontists/practitioners could more accurately determine where real change in attachment level has occurred, they may ultimately improve the diagnosis and treatment of periodontitis.

The rapid expansion and molecular outburst of periodontics is of little clinical interest and utility. There are several unanswered day to day clinical measurements that are basically considered for assessment of disease progression and periodontal treatment. If the clinical research concentrates on these basic clinical entities, the subject periodontics would scale better heights. By ignoring these problems and inclining towards molecular aspects, a great imbalance is created in understanding the growth of clinical periodontics.

A great deal of drugs and materials are used for treatment of periodontics. However, the clinical measurements are still confounded with problems. Solving these should be the major interest of the clinical periodontal research. Despite certain disadvantages of stent, the fixed reference point provides a simple solution for CAL measurement.
